# D-loop Mutations in Renal Cell Carcinoma Improve Predictive Accuracy for Cancer-Related Death by Integrating with Mutations in the *NADH Dehydrogenase Subunit 1* Gene

**DOI:** 10.3390/genes10120998

**Published:** 2019-12-02

**Authors:** Hakushi Kim, Tomoyoshi Komiyama, Masahiro Nitta, Yoshiaki Kawamura, Masanori Hasegawa, Sunao Shoji, Yasushi Orihashi, Chie Inomoto, Hiroshi Kajiwara, Naoya Nakamura, Hiroyuki Kobayashi, Akira Miyajima

**Affiliations:** 1 Department of Urology, Tokai University School of Medicine, Kanagawa, Isehara 259-1193, Japan; masan@tokai-u.jp (M.N.); kawausso@is.icc.u-tokai.ac.jp (Y.K.); hasem@sky.plala.or.jp (M.H.); sunashoj@mail.goo.ne.jp (S.S.); akiram@tokai.ac.jp (A.M.); 2 Department of Clinical Pharmacology, Tokai University School of Medicine, Kanagawa, Isehara 259-1193, Japan; komiyama@tokai-u.jp (T.K.); orihashi.y.c2@tsc.u-tokai.ac.jp (Y.O.); hkobayas@is.icc.u-tokai.ac.jp (H.K.); 3 Department of Pathology, Tokai University School of Medicine, Kanagawa, Isehara 259-1193, Japan; cisophia.ci@gmail.com (C.I.); h-kaji@ic.icc.u-tokai.ac.jp (H.K.); naoya@is.icc.u-tokai.ac.jp (N.N.)

**Keywords:** mitochondrial DNA, D-loop mutations, NADH dehydrogenase subunit 1, renal cell carcinoma, cancer-specific survival, prognostic factor

## Abstract

Renal cell carcinoma (RCC) is associated with various genetic alterations. Although whole-genome/exome sequencing analysis has revealed that nuclear genome alterations are associated with clinical outcomes, the association between nucleotide alterations in the mitochondrial genome and RCC clinical outcomes remains unclear. In this study, we analyzed somatic mutations in the mitochondrial D-loop region, using RCC samples from 61 consecutive patients with localized RCC. Moreover, we analyzed the relationship between D-loop mutations and *NADH dehydrogenase subunit 1* (*MT-ND1*) mutations, which we previously found to be associated with clinical outcomes in localized RCC. Among the 61 localized RCCs, 34 patients (55.7%) had at least one mitochondrial D-loop mutation. The number of D-loop mutations was associated with larger tumor diameter (>32 mm) and higher nuclear grade (≥ISUP grade 3). Moreover, patients with D-loop mutations showed no differences in cancer-specific survival when compared with patients without D-loop mutations. However, the co-occurrence of D-loop and *MT-ND1* mutations improved the predictive accuracy of cancer-related deaths among our cohort, increasing the concordance index (C-index) from 0.757 to 0.810. Thus, we found that D-loop mutations are associated with adverse pathological features in localized RCC and may improve predictive accuracy for cancer-specific deaths when combined with *MT-ND1* mutations.

## 1. Introduction

In renal cell carcinoma (RCC), representative gene mutations and chromosomal hyperploidy have been shown to depend on the histological subtype of the RCC, e.g., clear cell, papillary, chromophobe, or other subtypes [1]. Mutations in the *von Hippel–Lindau* gene have been extensively studied in clear cell RCC (ccRCC) and have been shown to play a critical role in the initiation and progression of this disease [2]. Papillary RCC is the second most common histological subtype of RCC and is characterized genetically by trisomy and tetrasomy of chromosome 7, trisomy of chromosome 17, and loss of the Y chromosome [3]. Chromophobe RCC accounts for approximately 5% of all RCCs and is characterized by loss of chromosomes 1, 2, 6, 10, 13, 17, and 21 [4,5]. Gene mutations in RCC do not only affect the tumor histological phenotype but also alter clinical behaviors or outcomes. Recent large-scale whole-exome and targeted sequencing studies have revealed frequent recurrent mutations in ccRCC [6,7] and have reported that specific gene mutations, such as *BAP1* or *SETD2* mutations, are associated with shorter overall survival and higher relapse rates [8]. A comprehensive genome analysis of RCC is currently underway to assess nuclear DNA (nDNA) mutations in RCC; however, the mutational profiles of mitochondrial DNA (mtDNA) in patients with RCC have not been sufficiently elucidated, and the associations of mtDNA mutations with clinicopathological features and prognoses among patients with localized RCC are poorly understood.

MtDNA is a circular, double-stranded DNA consisting of 16,569 bp and has a more compact DNA structure than nDNA. MtDNA has 13 protein-coding regions and a displacement loop (D-loop) region that controls the replication and transcription of mtDNA. In various types of cancers, somatic mutations in mtDNA were identified using tumor and paired nontumor tissues [9]. Moreover, several studies have reported that mtDNA mutations may have applications as prognostic markers [10,11]. In a previous study, we analyzed mutations in the mitochondrial NADH dehydrogenase subunit 1 (*MT-ND1*) gene to determine their associations with clinicopathological parameters and postoperative recurrence of RCC in 62 Japanese patients [12]. Our previous results suggested a significant association between the presence of *MT-ND1* mutations and the postoperative recurrence of localized RCC. Accordingly, mtDNA mutations may have applications in predicting malignant behaviors that cannot be explained by clinicopathological findings alone [12,13].

The D-loop region, a noncoding region of approximately 1120 bp that is responsible for the regulation of the replication and transcription of mtDNA, is a known mutational hot spot and accumulates mutations at higher rates than other coding regions of mtDNA [14,15,16,17]. The D-loop region has been reported to have a high mutation for various cancers, such as skin basal cell carcinoma, urothelial carcinoma, and colorectal carcinoma [18,19,20]. In addition, mutations in the D-loop region in cancer tissues are predictive of clinical outcome. The presence of D-loop mutations in pediatric acute leukemia, esophageal squamous cell carcinoma, oral squamous cell carcinoma, and hepatocellular carcinoma has been reported to be a risk factor for poor prognosis. Moreover, in RCC, a previous study reported a relationship between single nucleotide polymorphisms in the D-loop region and survival rates [21]. However, no studies have reported the association between somatic mutations in the D-loop region and cancer-specific survival (CSS).

Accordingly, in this study, we investigated mutation profiles of the D-loop region in RCC, the association of D-loop mutations with clinical outcomes, and the impact of D-loop mutations combined with *MT-ND1* mutations on predictive accuracy for clinical outcomes. Overall, we found that D-loop mutations are associated with adverse pathological features in localized RCC, which may improve the prediction of cancer-specific deaths when used in combination with *MT-ND1* mutations.

## 2. Materials and Methods 

### 2.1. Study Populations and Tissues

In total, 61 consecutive patients with localized RCC who underwent curative surgery (nephrectomy or partial nephrectomy) between January and December 2010 at a single institution were enrolled in this study. Demographic and pathological data were obtained from medical records and pathological reports. Formalin-fixed, paraffin-embedded (FFPE) tissue specimens were collected from these 61 patients. This study was approved by the institutional review board for clinical research of Tokai University (Approval No. 15R-065).

### 2.2. RCC Tissue Sampling

The sampling method for representative cancerous tissues and corresponding noncancerous renal tissues from FFPE tissue specimens was described previously [12]. Briefly, the locations of cancerous tissues and the corresponding noncancerous tissues within the FFPE tissue specimens were recorded by two pathologists (C.I. and H.Ka); subsequently, the tissues were obtained with an 18-G needle.

### 2.3. DNA Extraction, Polymerase Chain Reaction (PCR), and Sanger Sequencing

FFPE tissue punch samples were washed three times with 500 μL lemosol and three times with 99.5% ethanol to remove lemosol. Tissues were next incubated with 200 μg proteinase K in HMW Buffer (10 mM Tris-Cl [pH 8.0], 150 mM NaCl, 10 mM ethylenediaminetetraacetic acid [EDTA], 0.1% sodium dodecyl sulfate) at 60 °C overnight, followed by extraction with phenol–chloroform (phenol/chloroform/isoamyl alcohol, 25:24:1) twice at 11,000 rpm for 10 min at room temperature. DNA was precipitated by the addition of 0.1 volume of 3 M Na-AcOH and 2.5 volumes of ice-cold ethanol. After centrifugation at 15,000 rpm at 4 °C for 20 min, DNA pellets were rinsed with 70% cold ethanol, dried, and dissolved in TE buffer (10 mM Tris-HCl [pH 8.0] and 1 mM EDTA). Total mtDNA was amplified by PCR with the following primer sets, which were designed to cover the entire 1120-bp mtDNA D-loop region: HUMmt-148F, 5′-ATCCCATTATTTATCGCACCT-3′; homoR1, 5′-AAATAATAGGATGAGGCAGGAATCAAAGA-3′; Homo_mt_Gap_3F, 5′-TCGGAGGACAACCAGTAAGC-3′; Homo_mt_Gap_3R, 5′-GCACTCTTGTGCGGGATATT-3′; homoF2, 5′-GCACTCTTGTGCGGGATATT-3′; F376, 5′-TAACACCAGCCTAACCAGATTTC-3′; R505, 5′-TGTGTGTGCTGGGTAGGATGG-3′; F320, 5′-GCTTCTGGCCACAGCACTTAAAC-3′; R465, 5′-GATGAGATTAGTAGTATGGGAGTGGG-3′; F16137, 5′-CCATAAATACTTGACCACCTGTAG-3′; R16269, 5′-AGGTTTGTTGGTATCCTAGTGGGTGA-3′; F16137, 5′-CCATAAATACTTGACCACCTGTAG-3′; and R16231, 5′-GAGTTGCAGTTGATGTGTGATAGTTG-3′.

PCR was performed by KOD FX Neo (Toyobo, Osaka, Japan) under the following conditions: melting at 98 °C for 5 s, annealing at 63–66 °C for 15 s, and extension at 68 °C for 20 s, for a total of 35 cycles. PCR products were treated with EXOSAP-IT (Affimetrix, Santa Clara, CA, USA) and directly sequenced using a Big Dye Terminator v3.1 Reaction Kit (Applied Biosystems, Torrance, CA, USA) and an ABI 3500xL DNA sequencer (Life Technologies, Carlsbad, CA, USA). We also evaluated sequences in the D-loop region from one to five times to confirm the mutations. D-loop sequence data were assembled using ATCG software (Genetyx, Tokyo, Japan). Accession numbers for the nucleotide sequences of the D-loop obtained from 61 patients included in the present study are as follows: LC 491360–LC 491420.

### 2.4. Somatic Mutations in the D-loop Region

The alignment of the nucleotide sequence of the D-loop region was performed by Clustal W using Molecular Evolutionary Genetics Analysis (MEGA, version 6; Tempe, AZ, USA; Pennsylvania, USA; Tokyo, Japan) [22]. NC_012920.1 was selected as the reference sequence for the D-loop region. As previously described, the determination of somatic mutations in the D-loop region by comparative sequence analysis was divided into two steps [12]. Initially, we extracted the D-loop sequences of 61 healthy Japanese individuals from DDBJ/EMBL/GenBank databases and then extracted candidates for mutation sites in the D-loop region. Next, candidate mutation sites were compared with the sequence data of corresponding noncancerous renal tissue, and finally, somatic mutations in the RCC tissue were determined.

### 2.5. Somatic Mutations in the MT-ND1 Gene

*MT-ND1* sequence data for 61 Japanese patients with localized RCC were extracted from DDBJ/EMBL/GenBank databases, as described in our previous study [12]. Accession numbers were LC178840.1–LC178883.1 and LC178885.1–LC178901.1. Mutation sites were determined based on NC_012920.1 as the reference sequence for the *MT-ND1* gene.

### 2.6. Statistical Analysis

All statistical analyses were performed with JMP version 12.0.1 (SAS Institute, Cary, NC, USA), R (The R Foundation for Statistical Computing, Vienna, Austria), and EZR (Saitama Medical Center, Jichi Medical University, Saitama, Japan), a graphical user interface for R that adds statistical functions frequently used in biostatistics [23]. Seven demographic and pathological variables were selected to evaluate their associations with the presence/absence of D-loop mutations, the number of D-loop mutations, and CSS. Categorical variables were calculated using Fisher’s exact tests and Chi-square tests. Mann–Whitney U tests were used for continuous variables. CSS was calculated using the Kaplan–Meier method as the time from surgery to death caused by RCC, and results were compared using log-rank tests. Seven variables were binarized as follows: age (≤ 62 years versus > 62 years), sex (women versus men), tumor diameter (≤ 32 mm versus > 32 mm), histology type (ccRCC versus non-ccRCC), pT stage (≤ pT2 versus ≥ pT3), International Society of Urological Pathology (ISUP) grade (1/2 versus 3/4), and microvascular invasion (MVI) (absence or presence). Age and tumor diameter were separated using median values. The other variables were binarized according to our previous study [12]. We also evaluated the association of CSS with D-loop mutations with or without *MT-ND1* mutations identified in our previous study. The C-index was calculated to discriminate the predictive accuracy for CSS between a model, including only *MT-ND1* mutations and D-loop mutations added to the *MT-ND1* model [24]. The C-index ranged from 0 to 1.0, with 1.0 indicating a perfect model and 0.5 indicating a random model or no discrimination. Results with *p* values of less than 0.05 were considered statistically significant.

## 3. Results

### 3.1. Demographic and Pathological Features of the Study Population

The characteristics of the study population with and without D-loop mutations are shown in Table 1. Overall, the median age of patients was 62 years (range: 39–80 years). The cohort contained 47 men and 14 women and comprised 42 patients with nephrectomy and 19 patients with partial nephrectomy. The median observation period was 95 months (range: 29–112 months). As of August 2019, recurrence had occurred in 10 patients, and five patients had died of cancer-related causes. Pathological findings showed that the excised specimens had a median tumor diameter of 32 mm (range: 12–105 mm), and there were 54 patients with ccRCC and seven patients with RCC of other tissue types. The pathological T stage was T1 in 53 patients, T2 in three patients, T3 in four patients, and T4 in one patient. The nuclear grade was grade 1 in two patients, grade 2 in 50 patients, grade 3 in seven patients, and grade 4 in two patients, according to the International Society of Urological Pathology (ISUP) classification. Microvascular invasion (MVI) was observed in 13 patients. There were no significant differences in demographic and pathological data between patients with or without D-loop mutations.

### 3.2. Mitochondrial D-loop Mutations

In total, 79 mutations were identified at 51 sites in the D-loop region of 34 patients (55.7%, 34/61 patients). The mean and median number of mutation sites per patient were 1.3 and 1 (SD: 1.75, range: 0–8) sites, respectively, whereas the mean and median number of patients per mutation site was 1.5 and 1 (SD: 1.07, range: 1–7) patients, respectively. Specifically, patients hk370 and hk407 contained eight mutations in the D-loop region, which accounted for the highest number of mutations per patient. The mutation profiles of the D-loop region and *ND1* gene are shown in Table 2. The D-loop mutation rate was 1.295 (79 mutations/61 patients), and the *ND1* mutation rate, as determined in our previous study, was 0.475 (29 mutations/61 patients) [12]. The D-loop mutation rate was approximately 2.7 times higher than the *MT-ND1* mutation rate. The number of mutation sites per patient was associated with age (> 62 years), histological subtype (non-clear subtype), tumor diameter (> 32 mm), and ISUP grade (grades 3 and 4), as shown in Table 3. Of the 51 mutation sites, 17 mutation sites were found in more than two patients. Mutation at position 16295 was found in seven patients (hk362, 377, 379, 384, 407, 415, and 416), at position 249 in five patients (hk384, 388, 404, 413, and 418), and at position 310 in three patients (hk376, 400, and 403).

### 3.3. Survival Analysis for Integration of Mutations in the MT-ND1 Gene and D-loop Region

**Figure 1:**
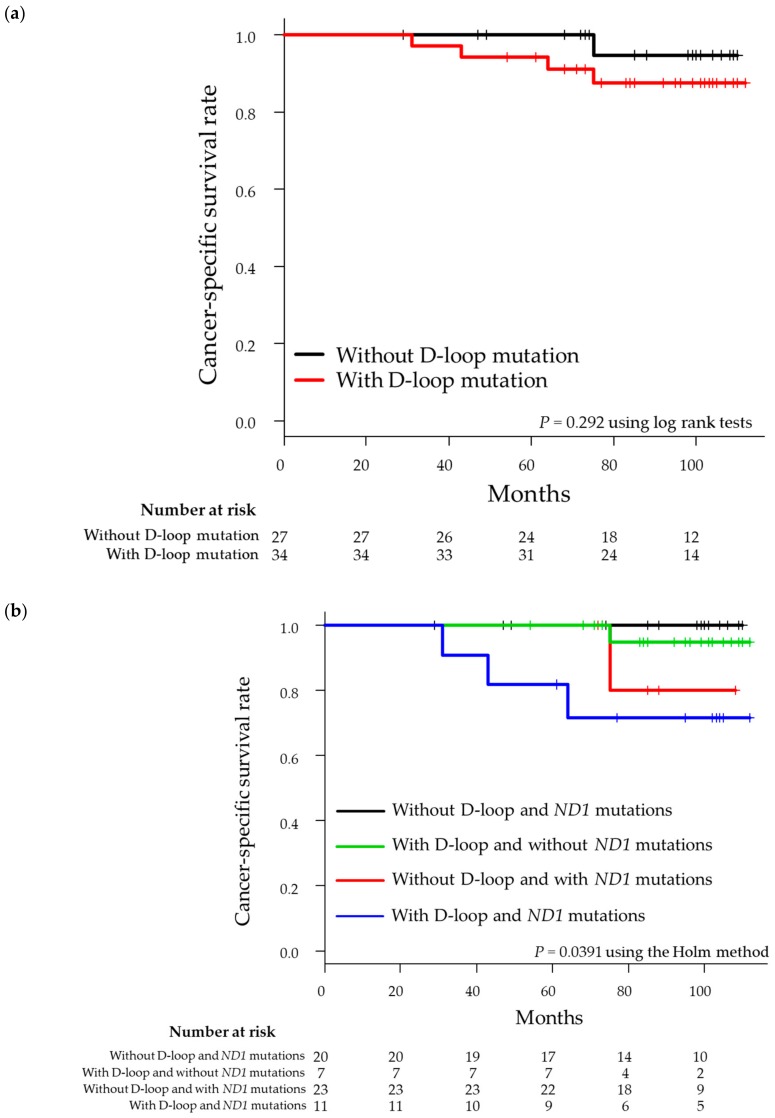
Kaplan–Meier plot of cancer-specific survival in 61 patients with localized renal cell carcinoma. (**a**) Patients were stratified into tumors with or without D-loop mutations. (**b**) Patients were stratified according to the presence of D-loop and/or *MT-ND1* mutations.

As shown in Figure 1a, CSS did not significantly differ according to the presence (94.1%) or absence of D-loop mutations (100%; *p* = 0.292). After the integration of mutation data for the D-loop region and *MT-ND1* gene, 41 patients were found to have mutations in the D-loop region and/or *MT-ND1* gene. Of these 41 patients, 11 patients had mutations in both the D-loop region and the *MT-ND1* gene, 23 patients had mutations only in the D-loop region, and seven patients had mutations only in the *MT-ND1* gene, whereas 20 patients did not have mutations in either the D-loop region or the *MT-ND1* gene. Log-rank tests further showed that CSS was worse in patients with both D-loop and *MT-ND1* mutations (Figure 1b). After integrating the data for D-loop mutations and *MT-ND1* mutations, we found that the concordance index (C-index) increased from 0.757 to 0.810 (Table 4).

### 3.4. Relationship Between Mutation Site and Other Cancers Based on Database Analysis

We focused on mutation sites from five patients (hk385, hk392, hk394, hk400, and hk403) who had died from RCC. Among these patients, three (hk385, hk392, and hk403) had mutations in both the D-loop region and *MT-ND1*. In addition, we found that patient hk394 had mutations only in the *MT-ND1* gene, whereas patient hk400 had mutations only in the D-loop region. Therefore, we examined related studies using the MITOMAP and PubMed (NCBI) databases to determine whether these mutation sites may be related to other types of malignant tumors as well (Table 5). We found that previous reports had described associations of mutations in the D-loop region with esophageal cancer, nasopharyngeal carcinoma, breast cancer, and ovarian cancer. Additional reports described associations between mutations in the *MT-ND1* gene and thyroid tumors, ovarian carcinoma, colorectal cancer, and prostate cancer.

## 4. Discussion

In the current study, we identified somatic D-loop mutations among 55.7% of patients with localized RCC. The mutation number per patient in the D-loop region was approximately 2.7-fold higher than that in the *MT-ND1* gene, as determined in our previous study [12]. A recent large-scale study analyzing the whole mitochondrial genome in prostate cancer using next-generation sequencing showed that the D-loop region was the most frequently mutated region, with mutations present in 15.4% of tumors [25]. However, no studies have evaluated the full range of sequences of the D-loop region from localized RCC tissue using formalin-fixed, paraffin-embedded (FFPE) specimens. To the best of our knowledge, this is the first study demonstrating a high mutation rate in the D-loop region in Japanese patients with localized RCC.

In our RCC cohort, larger tumor diameter (>32 mm) and higher ISUP grade (≥grade 3) were associated with larger numbers of D-loop mutations. Several previous studies have also reported associations between the number of D-loop mutations in various cancerous tissues and an adverse pathological state, such as tumor differentiation and advanced T-status [17,26]. These results suggested that the accumulation of mutations in the D-loop region may be related to an aggressive cancer phenotype. Combination therapy with immune checkpoint inhibitors has recently been recommended as first-line treatment by the International Metastatic RCC Database Consortium for patients with metastatic ccRCC with intermediate to poor risk [27,28]. Tumor mutation burden (TMB) is a known biomarker that serves as a predictive factor for the effectiveness of immune checkpoint inhibitors in multiple cancers [29,30,31]. Thus, mutations in the D-loop region, which occur more frequently than mutations in nDNA, may act in a similar way to TMB and function as potential biomarkers for predicting the effectiveness of immune checkpoint inhibitors.

The association between the presence of D-loop mutations and clinical prognosis is controversial. A recent study analyzing somatic mutations in the D-loop region of 120 patients with oral squamous cell carcinoma showed that the 5-year survival rate in patients with somatic mutations was significantly higher than that in patients without mutations [32]. Alternatively, the esophageal squamous cell carcinoma survival rate of patients with D310 mutations was lower than that in patients without this mutation [33]. In our RCC cohort, the presence of D-loop mutations was not significantly associated with CSS when considered alone; however, when integrating mutations in the *MT-ND1* gene and D-loop region, there was a clear separation of the CSS curve. Indeed, 11 patients with both *MT-ND1* and D-loop mutations showed worse CSS, and the 5-year CSS was 81.8%. Furthermore, our results revealed that the 5-year CSS rate in 20 patients without mutations in the D-loop region or *MT-ND1* gene was 100%. This result indicates that patients without any mutations in the *MT-ND1* gene and D-loop region may have a favorable prognosis.

Analysis of D-loop mutations, together with *MT-ND1* gene mutations, may improve the accuracy of predictive models for the CSS of localized RCC. To further examine the accuracy of these models, we evaluated the predictive power of each model by calculating the C-index [24]. The results showed that the C-index for predicting CSS improved from 0.757 to 0.810 by integrating *MT-ND1* mutations and D-loop mutations. To the best of our knowledge, only a few studies have previously reported the ability of mtDNA mutations to predict clinical outcome in patients with RCC [12,21,34]. Moreover, this is the first study to report the ability of D-loop mutations to improve the prediction accuracy for CSS in localized RCC.

Database analysis revealed that mutation sites related to poor prognosis in patients in this study were found in other types of malignant tumors, such as nasopharyngeal carcinoma, papillary thyroid carcinoma, and ovarian cancer. Thus, these findings suggested that these mutation sites may be candidates for predicting the survival of patients with localized RCC and other malignant tumors. Indeed, patients with mutations in both regions showed a higher risk of recurrence or death, and three patients (hk385, hk392, and hk403), who had both of these mutations, died during our study.

Our study had several limitations. First, the study was conducted at a single center, and patient data, including outcomes and pathological data, were collected retrospectively. Therefore, there may have been some bias to the results. In addition, our study cohort was small. Thus, further studies with larger cohorts and a multicenter center setting are needed. Second, we did not evaluate smoking or alcohol habits, which may affect mtDNA mutations. Moreover, we did not analyze nDNA mutations, which may be correlated with mtDNA for evaluating clinical outcomes.

## 5. Conclusions

The present study was retrospective in its design, and the study cohort was small; therefore, prospective and larger cohort studies are required to validate our results. Nevertheless, we showed that there were high mutation rates in the mitochondrial D-loop region and that accumulation of D-loop mutations was associated with adverse pathological tumor features in localized RCC. Furthermore, we found that patients with RCC with both D-loop and *MT-ND1* mutations exhibited more advanced CSS than patients with either D-loop mutations or *MT-ND1* mutations. The C-index improved following the inclusion of D-loop mutations in the ND1 risk model. Thus, we established an improved predictive model using genetic markers of mtDNA mutations. 

## Figures and Tables

**Table 1:** Characteristics of patients with localized renal cell carcinoma (RCC) with or without D-loop mutations.

Variable	Overall (*N* = 61)	With D-Loop Mutations (*N* = 34)	Without D-Loop Mutations (*N* = 27)	*p* Value
Age (years), median (range)	62 (39–80)	67 (39–80)	60 (39–79)	0.188
Sex				
Female	14	10 (16.4)	4 (6.6)	0.228
Male	47	24 (39.3)	23 (37.7)	
Histological subtype				
Clear cell subtype	54	28 (45.9)	26 (42.6)	0.121
Non clear subtype	7	6 (9.8)	1 (1.6)	
Tumor diameter (mm), median (range)	32 (12–105)	36.5 (13–100)	28 (12–105)	0.069
Pathological T stage				0.156
T1a	39	18	21	
T1b	14	11	3	
T2a	3	1	2	
T2b	0	0	0	
T3a	1	1	0	
T3b	3	2	1	
T4	1	1	0	
ISUP grade				0.156
1	2	1	1	
2	50	25	25	
3	7	6	1	
4	2	2	0	
MVI				
Absence	48	25 (41.0)	23 (37.7)	0.352
Presence	13	9 (14.8)	4 (6.6)	

RCC, renal cell carcinoma; MVI, microvascular invasion.

**Table 2:** Mutation profiles of the D-loop region and the *NADH dehydrogenase subunit 1 (MT-ND1)* gene.

Patient	Base Change in the D-Loop	Number of Mutations	Base Change in MT-ND1	Number of Mutations	Sum of Mutations
hk346	None	0	None	0	0
hk347	None	0	C4197Y	2	2
			T4248Y		
hk348	None	0	None	0	0
hk350	C16260Y	1	None	0	1
hk352	None	0	None	0	0
hk354	C146Y	2	None	0	2
	T152Y				
hk355	A16183C	4	C3497T	1	5
	T16189Y				
	T16217Y				
	T16223Y				
hk356	None	0	None	0	0
hk357	T16189Y	1	C4197Y	1	2
hk358	None	0	None	0	0
hk359	G94R	1	None	0	1
hk361	C146Y	3	None	0	3
	T152Y				
	C16261Y				
hk362	T16209Y	2	C3970Y	1	3
	C16291Y				
hk363	None	0	T4248Y	1	1
hk364	C16527T	1	C3497T	2	3
			G3635A		
hk365	None	0	None	0	0
hk366	None	0	None	0	0
hk367	G16390R	1	None	0	1
hk368	None	0	C4197Y	2	2
			T4248Y		
hk369	C16290Y	2	None	0	2
	G16319R				
hk370	A202R	8	None	0	8
	309insCC				
	T16136C				
	A16183C				
	T16217Y				
	T16223Y				
	C16261Y				
	C16527T				
hk371	None	0	None	0	0
hk372	T16311Y	2	A4200W	2	4
	A16316R		T4216Y		
hk374	None	0	None	0	0
hk375	None	0	None	0	0
hk376	8C 303 2C	2	None	0	2
	T310Y				
hk377	T60W	3	None	0	3
	C16261T				
	T16368C				
hk379	C16291T	1	None	0	1
hk380	None	0	None	0	0
hk382	None	0	A4200W	2	2
			T4216Y		
hk383	None	0	C3572ins	1	1
hk384	249Adel	3	None	0	3
	A16203G				
	C16291Y				
hk385	G68A	2	G3496T	3	5
	G16390R		C4141Y		
			T4248Y		
hk387	None	0	C4197Y	2	2
			T4248Y		
hk388	249Adel	3	None	0	3
	T485C				
	C16344Y				
hk389	C16245Y	1	None	0	1
hk390	None	0	None	0	0
hk391	None	0	None	0	0
hk392	T16195C	3	G4048R	2	5
	T16297C		C4071Y		
	T16298C				
hk393	T60C	2	T3368Y	1	3
	G263A				
hk394	None	0	A4200W	2	2
			T4216Y		
hk395	None	0	None	0	0
hk397	None	0	None	0	0
hk398	None	0	None	0	0
hk400	T72Y	2	None	0	2
	T310Y				
hk401	A202G	1	T4117Y	1	2
hk402	None	0	None	0	0
hk403	G73R	4	C3328Y	2	6
	C194Y		C3970T		
	T204C				
	T310Y				
hk404	249Adel	2	None	0	2
	A16203G				
hk405	C16320A	1	G4113R	1	2
hk407	G73A	8	None	0	8
	T131C				
	C16111Y				
	T16140Y				
	C16234Y				
	T16243Y				
	C16291Y				
	A16463G				
hk408	None	0	None	0	0
hk410	C6 568 C8	1	None	0	1
hk411	G251R	2	None	0	2
	C194Y				
hk412	None	0	None	0	0
hk413	249Adel	2	None	0	2
	T16172Y				
hk414	None	0	None	0	0
hk415	C16291T	1	None	0	1
hk416	C16291Y	1	None	0	1
hk417	A16254G	1	None	0	1
hk418	249Adel	5	None	0	5
	G16129R				
	C16232M				
	T16249Y				
	C16344Y				

**Table 3:** Association between the number of D-loop mutations and demographic/pathological factors.

Variables	Overall (*N* = 61)	Number of D-Loop Mutations Median (IQR)	*p* Value
Age (years)			0.012
≤62	31	0 (0–1)	
>62	30	1.5 (0–2.75)	
Sex			0.311
Female	14	1 (0.25–2)	
male	47	1 (0–2)	
Histological subtype			0.045
Clear cell subtype	54	1 (0–2)	
Non-clear cell subtype	7	2 (1–4)	
Tumor diameter			0.029
≤32	31	0 (0–1.5)	
>32	30	1 (0.25–2.0)	
Pathological T stage (pT)			0.197
≤pT2	56	1 (0–2)	
≥pT3	5	2 (1–3)	
ISUP grade			0.029
≤ISUP2	52	0.5 (0–2)	
≥ISUP3	9	2.0 (1–3)	
MVI			0.257
Absencee	48	1 (0–2)	
Presence	13	1 (0–2)	

**Table 4:** C-index of a predictive model for CSS.

Model	C-Index	Lower	Upper
		95% CI	95% CI
ND1	0.757	0.419	1.000
ND1 and D-loop	0.810	0.527	1.000

**Table 5:** Database analysis of specific mutation sites in the D-loop region and *MT-ND1* gene in five patients who died from RCC.

Patient	D-Loop			*MT-ND1*			Sites of Recurrence
	Our study Polymorphisms	Cancer Types	MITOMAP Polymorphisms	Our study Polymorphisms	Cancer Types	MITOMAP Polymorphisms	Cancer Types
hk385	G68A	Esophageal cancer PMID: 14639607	G68A	T4248Y	Thyroid tumors PMID: 10803467	T4248C	Liver Lung
	G16390R	1. Nasopharyngeal carcinoma PMID: 18376149	G16390A	None	None		
		2. Breast cancer PMID: 16568452	G16390 A/G				
		3. Ovarian cancer PMID: 18842121	G16390A				
hk392	T16195C	None		G4048R	1. Ovarian carcinoma PMID: 11507041	G4048A	Local Lung
					2. Colon cancer PMID: 29842994	G4048A	
	T16297C	Nasopharyngeal carcinoma (PMID: 18376149)	T16297C	C4071Y	Ovarian carcinoma PMID: 11507041	C4071T	
	T16298C	1. Nasopharyngeal carcinoma PMID: 18376149	T16298C	None	None		
		2. Ovarian cancer PMID: 18842121	T16298C				
hk394	None	None		T4216Y	1. Thyroid tumors PMID: 10803467	T4216C	Lung
					2. Prostate cancer PMID: 11526508	T4216C	
					3. Colorectal cancer PMID: 19050702	T4216C	
hk400	T72Y	Ovarian cancer PMID: 18842121	T72C	None	None		Bone Local Lung
	T310Y	Nasopharyngeal carcinoma PMID: 18376149	T310C				
hk403	A73R	1. Ovarian cancer PMID: 16942794, 18842121	A73G	C3970T	1. Ovarian carcinoma PMID: 11507041	C3970T	Bone
		2. Nasopharyngeal carcinoma PMID: 18376149	A73G				
					2. Colorectal cancer PMID: 19050702	C3970T	
